# Mapping the social determinants of substance use for pregnant-involved young Aboriginal women

**DOI:** 10.1080/17482631.2016.1275155

**Published:** 2017-01-23

**Authors:** Sana Z. Shahram, Joan L. Bottorff, Nelly D. Oelke, Donna L. M. Kurtz, Victoria Thomas, Patricia M. Spittal, and For the Cedar Project Partnership>

**Affiliations:** ^a^ Faculty of Health and Social Development, University of British Columbia, Kelowna, Canada; ^b^ Institute for Healthy Living and Chronic Disease Prevention, and School of Nursing, Faculty of Health and Social Development, University of British Columbia, Kelowna, Canada; ^c^ Faculty of Health Sciences, Australian Catholic University, Melbourne, Australia; ^d^ School of Nursing, Faculty of Health and Social Development, University of British Columbia, Kelowna, Canada; ^e^ Wuikinuxv Nation, The Cedar Project, Vancouver, Canada; ^f^ School of Population and Public Health, University of British Columbia, Vancouver, Canada

## Abstract

There is a dearth of knowledge about the social determinants of substance use among young pregnant-involved Indigenous women in Canada from their perspectives. As part of life history interviews, 17 young pregnant-involved Indigenous women with experiences with substances completed a participant-generated mapping activity CIRCLES (Charting Intersectional Relationships in the Context of Life). As women created their maps, they discussed how different social determinants impacted their experiences with pregnancy and substance use. The social determinants identified and used by women to explain determinants of their substance use were grouped into 10 themes: traumatic life histories; socioeconomic status; culture, identity and spirituality; shame and guilt; mental wellness; family connections; romantic and platonic relationships; strength and hope; mothering; and the intersections of determinants. We conclude that understanding the context and social determinants of substance use from a woman-informed perspective is paramount to informing effective and appropriate programs to support young Indigenous women who use substances.

## Background

Social determinants of health are key considerations in understanding health inequities observed in society (Halseth, 2013; Reading & Wien, 2009). These determinants typically include early child development, education, income, employment, the nature of social and physical environments, personal health practices and coping skills, access to health services, racism and gender (Halseth, 2013; Reading & Wien, 2009). In addition, Aboriginal^1^ ^,^ ^2^-specific determinants have been identified and include the social, political and historical contexts that also contribute to contemporary Aboriginal health, subsequent to and resulting from the practices of colonialism (Reading & Wien, 2009).

While researchers have begun to use social determinants in their approach to understanding addictions issues, there are gaps in the literature in relation to Indigenous^3^- specific social determinants and their impact on substance use. In a recent systematic literature review of peer-reviewed empirical research on the social determinants of substance use among Aboriginal women between 1999 and 2012, only 16 articles were identified (Shahram, 2016). The review revealed that the almost exclusive use of quantitative methods and the prioritization of certain social determinants of health over others have prevented a comprehensive and contextual understanding of substance use among Aboriginal women. Women’s perspectives on experiences that shape their substance use were largely missing from the literature.

Aboriginal young women constitute a group of women who are disproportionately affected by substance use in Canada and elsewhere, with a paucity of research examining their particular social contexts and drivers of use, particularly as it relates to pregnancy (Shahram, 2016; CARBC, 2008; Niccols, Dell, & Clarke, 2010). On account of imposed colonial conditions, Indigenous women in Canada are often living in high risk environments and are subsequently at particularly high risk for substance use. These inequities have consistently been linked to the social and cultural disruption and historical and intergenerational trauma that have characterized the experiences of Aboriginal people in Canada (Haskell & Randall, 2009; Mitchell & Maracle, 2005). Indigenous populations in Australia, New Zealand and the USA are considered to be most comparable to Aboriginal people in Canada, with similar disparities in health status (including maternal health issues), due, in part, to their collectively similar colonial histories (Bowen et al., 2014; Chamberlain et al., 2013). It has been suggested that Indigenous women who use substances may be particularly disadvantaged in facing triple marginalization, due to discrimination based on both gender and race (Pearce, Pederson, & Greaves, 2007), compounded by further marginalization as substance users (Brunen & Northern, 2000).

In addition, current social determinants of health models developed by researchers (Marmot, Friel, Bell, Houweling, & Taylor, 2008; Mikkonen & Raphael, 2010; Reading & Wien, 2009) fall short in capturing the specific and unique contexts of substance use among pregnant-involved young Indigenous women. Specifically, current models do not fully account for gender-related factors, the unique social conditions that underlie substance use among pregnant-involved young Indigenous women, or their intersectional relationships. Similarly, current models do not account for determinants that can support women’s wellness, resilience and strengths. Women’s experiences and their perspectives on factors influencing their experiences with both pregnancy and substance use, therefore, could inform further social determinants of health model development to begin to address these gaps.

There is a need to understand the social determinants of substance use among pregnant-involved young Indigenous women from women’s perspectives to inform strategies to support women’s strengths and wellness. The objective of this research project was therefore to understand how pregnant-involved young Indigenous women conceptualize and understand the social determinants of substance use and their intersections.

### Theoretical approach

This research was conducted in western Canada and was therefore informed by theoretical understandings of the cumulative effects of colonization (Hackett, 2005; Halseth, 2013; Hunting & Browne, 2012; Reading & Wien, 2009). Intergenerational traumas, systemic discrimination and displacement are the downstream effects of colonization, and experiences of racialization, discrimination, poverty and interpersonal violence often define the life experiences of Aboriginal women who use substances (Hunting & Browne, 2012). Postcolonial perspectives provide both the analytical framework and vocabulary for understanding how the socio-historical-political contexts contribute to the health, healing and human suffering of the research participants (Browne, Smye, & Varcoe, 2005).

To fully consider and analyze the context and influence of social power inequities, an intersectionality approach that avoids the use of additive lists in favor of focusing on the fluid and interactive nature of multi-level complex processes and systems that shape health inequities is necessary (Hankivsky, 2012). An intersectionality approach, informed by a post-colonial perspective, provides direction for conceptualizing the contributions of the social determinants of health to the issue of substance use, without sacrificing the complexity and fluidity of these moving parts for the sake of research ease.

## Methods

As part of a qualitative study exploring women’s experiences related to substance use and pregnancy (Shahram et al., 2017), a participant-generated mapping activity called CIRCLES (Charting Intersectional Relationships in the Context of Life Experiences with Substances) was completed, and the findings from this portion of the research are reported here. This research and the study protocol were approved by the Behavioural Research Ethics Board at the University of British Columbia and the Cedar Project Partnership that oversees research conducted with Cedar Project participants. This study was conducted with special attention to Chapter 9 of the Tri-Council policy statement *Ethical Conduct for Research Involving Humans*, which also guides the Cedar Project's research activities (Canadian Institutes of Health Research, Natural Sciences and Engineering Research Council of Canada, & Social Sciences and Humanities Research Council of Canada, 2014). . This research project was conducted with mentoring from experienced Cedar Project researchers, as well as in collaboration with the Cedar Project Partnership. The Partnership approved the conception, design and implementation of this research project, and ensured community representation throughout the research process. Additionally, by explicitly focusing on the strengths-based narratives of resilience and survival that characterize women’s lives, this research project contributes a counter-narrative to the often pathologizing and stigmatizing presentations of Indigenous mothers that dominate research findings and mainstream society, today. Women’s perspectives and voices have been prioritized as necessary contributions to extend the boundaries of knowledge in this field.

### Sample

Convenience sampling methods were used to recruit participants from the Cedar Project (Spittal et al., 2012), an ongoing longitudinal study exploring HIV- and hepatitis-C-related vulnerabilities among young Aboriginal men and women in three cities in western Canada. Recruitment occurred at all three study sites. Eligibility criteria included being an Aboriginal woman and being a current participant of the Cedar Project. Only pregnant-involved women, defined as having at least one experience of pregnancy between 14 and 30 years of age (in order to capture the experiences of young, pregnant women), were eligible to participate. Not restricting participation to only pregnant women provided the opportunity to explore women’s experiences before, during and after pregnancy to inform a deeper and more nuanced understanding of their experiences with substance use throughout their lives, as well as during pregnancy.

### Data collection

Seventeen women participated in an open-ended interview where they were invited to generate a map of what they considered to be important influences related to their substance use experiences. Participant-generated maps are a form of graphical elicitation techniques to collect data on relationships and complex ideas. Graphical and/or visual elicitation techniques involve mapping or diagramming data collection methods in conjunction with in-depth interviews to allow participants to reflect upon and visually depict complex thoughts more clearly and deeply than with interviews alone, in order to create concise visual snapshots of the complex resulting data for means of data analysis and reporting (Benninger & Savahl, 2016; Copeland & Agosto, 2012; Michaelson, McKerron, & Davison, 2015; Umoquit, Tso, Burchett, & Dobrow, 2011; Wheeldon & Faubert, 2009). Graphical elicitation techniques are also less constrained by language, dialect and literacy, and are less burdened by western psychological, biomedical or religious ideologies and biases (Chase, Mignone, & Diffey, 2010).

By working with an Elder and people who knew the participants well, efforts to establish trust with participants as well as assurances of confidentiality were used to facilitate data collection and to support interpretation of the results. The credibility of the qualitative findings was ensured through several strategies. When women returned for their follow-up interview, they were provided with a summary of their first interview. At this point, they were able to correct any mistakes and add or remove any information they felt pertinent. At this time, the primary researcher (S.Z.S) was also able to clarify any confusion she may have had with understanding the woman’s story. Additionally, the CIRCLES mapping exercise doubled as a member checking exercise. As women contemplated using the provided buttons (small round plastic discs on which the different determinants were written on) the researcher had generated from her interpretation of the woman’s first interview, they also commented on whether that was an accurate interpretation/influence in her life. Further to this, the analysis and findings were shared with the Cedar Project Partnership as well as with the researcher’s Indigenous mentor for feedback and input.

The primary researcher (S.Z.S) is not of Aboriginal-descent and this may have had an impact on the data collection and analysis process. (S.Z.S) is a woman and a visible minority, so this may have made her more aware of and more able to identify stories of oppression based on sexisms or racisms based on her own experiences. Every effort was made to prioritize women’s opinions and voices; however the CIRCLES maps must be understood as having been collaboratively created by women and the researcher.

#### CIRCLES data collection tool

**Figure 1.**
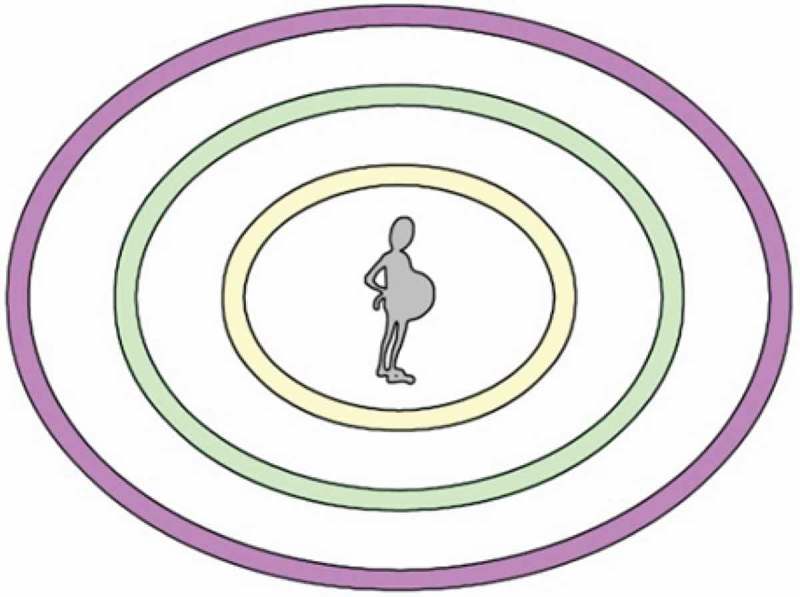
Blank CIRCLES map.

To support women’s use of graphical elicitation techniques, for the purpose of this study the CIRCLES map was designed by one of the researchers (SS) based on the Integrated Life Course and Social Determinants of Aboriginal Health Model [ILCSD] (Reading & Wien, 2009). As shown in Figure 1, the CIRCLES map included three concentric rings to reflect distal, intermediate and proximal determinants of substance use.

The CIRCLE map and a variety of buttons were provided to each woman to assist them in generating a map of important influences related to their substance use. Based on the participant’s first interview, 20 grey buttons were prepared with determinants the researcher heard the participant discuss in her first interview; 20 blue buttons were prepared with determinants other participants had used or that previous research had found to be important; and 20 blank pink buttons were provided for the participant to complete herself, should she want to add other items.

Participants were invited to use as many or as few buttons as they wanted while creating their map, and to add their own buttons as necessary. Participants were also informed that this was their own personal map, so there was no wrong way of creating it and they should trust their instincts on what made sense to them. Also, participants were encouraged to place buttons next to other buttons where they thought there was a relationship between those determinants. Women were encouraged to talk about the positioning of various buttons as they mapped their experiences. Once they had completed their CIRCLES maps, women were asked what they would want other people to understand about their lives based on the map they designed. Interviews were audio recorded and a photograph was taken of participants’ final maps.

### Data analysis

Data analysis was inductive and non-linear (Roper & Shapira, 2000). All interview data were transcribed verbatim and managed using computer software program, NVivo 10^TM^. Initially, several close reviews of the entire dataset were conducted to identify important or interesting features of the data. Transcripts were also read closely for explicit or implicit ways women constructed determinants in the lives and how they positioned them in relation to their current situation and future aspirations. Particular attention was given to the determinants (labeled buttons) women used and where they were placed on the map in relation to other buttons, and to which concentric circle they placed them on. An initial coding framework was developed by (S.Z.S) based on these initial reviews of the entire data set to begin to organize the data into meaningful categories. The transcripts were coded using this framework and codes were examined to find common patterns in women’s responses and to identify trends across all cases as well as alternatives that deviated from these dominant patterns. The coding categories were further developed and refined in collaboration with the entire research team in order to fully capture emerging themes and ensure credibility of the findings. During this process, some categories were collapsed and labels refined to more clearly capture identified themes. Data analysis concluded when categories were saturated and themes clearly defined.

The determinants identified by women were grouped into 10 themes. In addition, since emotional responses to identified determinants were a focus of women’s discussions as they completed the maps, these data were also coded and examined as consequences of, or in relation to, other social determinants in their lives.

Finally, in an effort to honor the Cedar Project Partnership’s priority to focus on strength and wellness models of health, the First Nations Health Authority in British Columbia’s concept of wellness (depicted visually in their Perspectives of Wellness) (First Nations Health Authority, 2011) was used as a lens in the interpretation of women’s maps. Pseudonyms were assigned to participants.

## Findings

**Figure 2.**
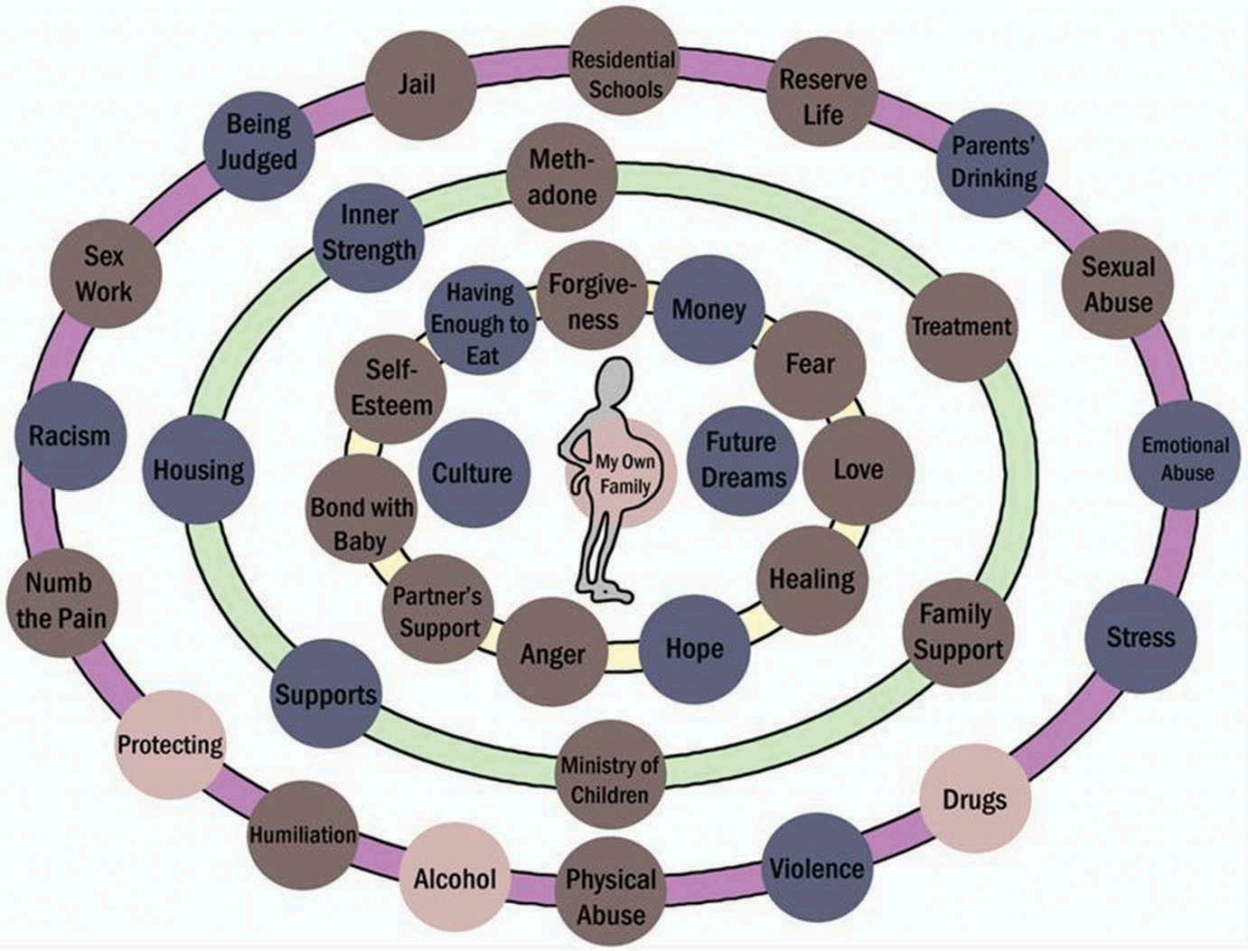
An example of a completed CIRCLES map. Note: Gray buttons were drawn from the participant’s own life history interview, blue buttons were drawn from other participants’ life history interviews and pink buttons were generated during the mapping activity.

The determinants used by women in their maps were grouped into 10 themes: traumatic life histories; socioeconomic status; culture, identity and spirituality; shame and guilt; mental wellness; family connections; romantic and platonic relationships; strength and hope; mothering; and, the intersections of determinants. Figure 2 shows an example of a completed CIRCLES map.

### Traumatic life histories

Issues related to traumatic life experiences were mostly mapped as distal determinants using buttons labeled “trauma”, “abuse” and/or “violence” to represent major detractors to overall wellness. For many women, these terms were not specific enough and they often used multiple and specific buttons, such as “tough love”, “sexual abuse”, “physical abuse” and “emotional abuse” to detail the cumulative and interrelated impact of multiple types of traumatic experiences to explain their use of substances to cope with these difficult experiences. Women also recognized that using substances as a coping mechanism often led to further traumatic experiences and that these repeated and continued experiences throughout their lives had devastating impacts on their sense of safety, hope and self-worth. When women looked at their final maps, they most often expressed a sense of surprise at seeing the multiple types of trauma they had experienced, survived and, in some cases, healed from.

### Socioeconomic status

Women placed buttons related to lacking access to money, education, adequate housing and food in various positions on their maps. However, regardless of where they placed these buttons, women discussed their low-socioeconomic status as something that consistently either exacerbated or contributed to other negative experiences in their lives, including the harms associated with substance use. For example, one participant stated:

Money should be here, here and here! [pointing at each of the three circles on the map] [laughs] I was always trying to get money, always. If not for me and my sisters and my mom, then for the drugs and alcohol or then when I had my kids and was sobered up, I was still trying to get money and feed my kids and clothe their backs. Ya [it goes] everywhere. It is a big thing. We struggled all our lives. (Sophie, mother of three, in her early-30s)

Another woman recounted that she spent most of her life trying to figure out how to have enough to eat. Although women were resourceful in supplementing their limited resources through support from family, partners or friends, local programs using food vouchers and dividing food into smaller portions, taking advantage of shelters and temporary housing, and securing money through survival sex work, dealing drugs and theft, women recognized that securing adequate resources dominated the majority of their time while also, in some cases, compromising their own health and wellness. A high priority for many women was getting a “job” (usually mapped as a proximal determinant) as a source of both income and “pride” and to secure economic stability for themselves and their families and to prevent apprehension of their children.

Dealing with these various resource-based issues from a young age also interfered with women’s education. While attending college or technical school was stressed as one of the happiest experiences in three women’s lives, most expressed regret that they had not completed their education. The “school” button was most often placed on the middle circle maps, either as previous, current or future support for women’s wellness as well as to inspire their children.

### Culture, identity and spirituality

Many of the women linked the absence of early cultural connections with substance use using the “culture” button on their maps to capture an important and complicated determinant in their lives. Most women expressed regret that traditional teachings and cultural activities had not been a bigger part of their lives when growing up and associated this with a lack of belonging and their search for approval and acceptance. For example, Sarah (mother of four, in her mid-thirties) who was completing her college education at the time of the interview, stated:

I struggled with like who I was, my identity because I was bounced around so much … my identity was a big one for me. That’s what affected like everything probably in this here [middle and inner circle]. I just couldn’t figure out where I belong so I just drank because I didn’t really care about too much. And that had to do with like residential [school] so … the more I learnt about it and the more I go to like, with all the workshops they have going on, and hearing everybody’s stories and just knowing … has helped.

Women also associated culture with spirituality and mapped spirituality-related buttons as a distal determinant to substance use. For women who acknowledged their belief in God, they shared that they found faith-based programs like Narcotics Anonymous and Alcoholics Anonymous particularly supportive. For other women, traditional healing practices were stressed as important to their spiritual wellness, as well as to their overall health.

Still, as women reflected on how to map buttons related to culture and spirituality, the discussion often turned to racism and identity. One woman stated simply: I was always ashamed of being Native”, reflecting the complex internalization of popular, and negative, representations of Indigenous peoples in Canada. For others, everyday experiences of racism were more blatant and these women expressed feeling angry, for example, when they were followed around in retail stores.

### Shame and guilt

While creating their maps, many women tried to make sense of their feelings of guilt and shame as they situated the buttons with these words on their maps, most often in the proximal circle, and to understand how these feelings were related to social determinants in their lives. While creating her map, Roxanne, a mother of two in her mid-thirties) who was trying to understand why she had not yet quit using drugs, contemplated the role of shame in her life:

I’m ashamed that I’m not doing it on my own and I’m just saying this out loud ‘cause I wanna know … is this why? I mean there are people there for me. Like in my building there’s … I wanna move out of there, I have no business being there. And why don’t I go and see [the support worker]? Being embarrassed that I can’t do it on my own, I guess.

As other women mapped words related to shame and guilt, they discussed how being able to reach out and ask for help would be a first important step towards their healing, but that feelings of inadequacy and being a burden on others often prevented them from doing so.

Low self-esteem was closely related to feelings of “shame” and “guilt” for many women and further impacted their emotional wellness. Women discussed how these feelings resulted from internalizing other peoples’ cruel words or actions towards them and how actively working to reverse these internalizations was an important support for their wellness. Women explained that “feeling judged” or “not welcome” were the main deterrents to accessing community supports, while some women discussed not wanting to access treatment because they either felt shame about previously attending and relapsing or that they were fearful of failure. Other barriers to attending treatment were the restrictions on their interactions during treatment and thoughts that quitting while away from “real life” was not going to be helpful in the long run. A major support for many women for dealing with feelings of shame and guilt was the opportunity to help other people, as Sarah explained while discussing her hopes for her future:

[I hope] that I can be a really strong role model for my family and for whoever that is struggling with any kind of addictions. I wouldn’t mind [working in] addictions … and being a good sober friend …. I love helping people.

Women’s inner strength was consistently apparent in these discussions as they explained the multiple coping strategies they employed to deal with these feelings on a daily basis including avoiding new or different environments as a protection mechanism, journaling, therapy, substance use treatment, talking about their feelings and sharing their story with others.

### Mental wellness

All of the women discussed the importance of their mental wellness to support their roles as mothers and their overall health. The majority of women placed buttons representing mental health-related determinants (e.g., “stress”, “fear”, “grief”) in the middle circle to demonstrate supporters and detractors of their mental health and substance use. As women placed the button “stress” on their maps, they discussed a variety of sources in their lives ranging from stress related to juggling their multiple roles, to dealing with their daily needs like securing somewhere to sleep, food to eat and supporting their partners; dealing with state intervention; and dealing with their addiction. Women also used buttons like “fear”, “confusion”, “pressure” and “anger” to represent feelings that detracted from their mental wellness. For many women, “grief” and “loss of a loved one” characterized much of their lives, and as they mapped these buttons they discussed how failing to deal with these issues had serious, negative repercussions for their mental wellness, their substance use and their overall health.

All women described their drug and alcohol use as way to “numb the pain” and loss in their lives. After having numbed or blocked out pain for many years, regaining sobriety, women explained, also meant coming face-to-face with memories or experiences that they were “in denial” about. However, many women stressed that coming to terms with what they had experienced and survived was an essential, albeit difficult, step of achieving mental wellness:

It’s good. I’m still working on it, you know what I mean? Ya, I’m striving for something. I want something good and it’s just stressful because it’s hard to get. Ya. Cause like, [now] I feel it [all]. [Including the good stuff], I never have to numb that. (Sophie)

Five women had been diagnosed with mental health conditions including depression, anxiety, an eating disorder and bipolar disorder and they created buttons to map these as either distal, intermediate or proximal determinants in their lives. Women discussed how these conditions were often further exacerbated by previous and current experiences of trauma and the high demands on their day-to-day lives. Women also identified supports for their mental wellness, often mapping buttons in the middle circle, including: “strength”, “safety” and “sharing my story”. They also discussed the importance of learning new coping techniques, healing from and forgiving past abuses, learning about their mental health issues and ways to support their own wellness as important supports for their mental wellness.

### Family connections

Family was a central focus of all women’s life experiences, and their subsequent substance use and pregnancy experiences, throughout the mapping exercise. Many women struggled with how to map their family’s influence in their lives, as it was often both positive and negative. While some explicitly mapped buttons with value-laden words like “family support” or “family enablers”, others used a button with just the word “family” to accommodate both the positive and negative influences of their family connections. The majority of women mapped words related to their families of origin in the middle or outer circle, and then mapped their children and partner in the inner circle. However, this was likely due to the fact that for many women, their maps were constructed to include an element of temporality and therefore their families of origin were considered to be removed or prior to their family consisting of their own children. Family was primarily mapped in the outer circle by women who were estranged from their families or saw them as having impacted either positively or negatively on everything else in their lives. For women who mapped family in the middle circle, they saw them as either a support or detractor of their overall wellness, with most of them seeing family as a source of both.

For example, family was a source of constant support in women’s lives, especially their mothers, from whom they often sought advice in good and bad situations. The support was also mutual, with many women describing their roles caring for or supporting their families. However, they also discussed how residential schooling, intergenerational perpetuation of harm, involvement in the foster care system and experiences of abuse and neglect detracted from their ability to form and maintain healthy family connections. Constant or permanent separation from their families left some women desperate for connections. Belinda, a mother of three, in her early-thirties who had been adopted from birth with her two older sisters, explained that her first times of trying alcohol, drugs and survival sex work were her efforts to feel connected with her older sisters (who were participating in those activities) and to have the “same feelings” as them.

“Foster care” involvement and being apprehended and separated from family was constructed and mapped as an important contributor to substance use. Not only were these experiences described as being extremely traumatic, but for some women this is when they were first introduced to alcohol and drugs. Regardless of their childhood experiences, forgiveness, reconnection and bonding with family members were identified as supporting their wellness, healing and recovery and, as such, figured prominently on women’s maps.

### Romantic and platonic relationships

Relationships played a large role in women’s substance use. They found it hard to quit or reduce use when their partner was still using drugs or alcohol or when they were abusive. In these cases, women mapped terms like “partner’s influence”, “partner’s drug use” or simply “partner” in the middle circle to accommodate their conflicted feelings towards their partners, hom they loved, but who also caused them harm. Women also often mapped these terms close to abuse terms in their outer circles as they discussed the cycle of violence in their lives. As Francesca, a mother of two, in her mid-twenties who was pregnant at the time of interview, explained: “I never knew a relationship without getting hit … everyone gets hit and it’s like another day.”

In contrast, women who were in positive relationships mapped “partner” in their inner circle, as well as “partner’s support” in the middle circle, reflecting their beliefs that maintaining healthy relationships was a source of wellness in their lives. These women described their relationships with their partners as being mutually supportive, especially in terms of quitting alcohol and drug use. In addition, several women discussed how having peer support from someone who had been through similar experiences and who could “relate to” them was an integral part of getting them into treatment to begin their healing. For women who had connected with resources, they highlighted services that provided tangible and practical resources, as well as knowledge, to support them in their daily lives as mothers in a non-judgemental, harm-reduction environment, as an important source of support. As Linda, in her early-twenties a new mother of one described, emotional support was an important need for many women:

If it wasn’t for the people at the [support centre], I wouldn’t have been able to do anything. They just loved coming over. They were his first aunties. Oh, they just came over to hold him. But, if I need anything I just gotta ask them and if they don’t know or if they can’t do it, then they direct me to someone who can … they’re still in his life. I still go over and visit.

Relationships were also embedded in communities, where drugs were easily available and socially promoted. While women felt accepted and safe in these communities surrounded by close friends and families, they also recognized that their connectedness in these communities was a main obstacle in reducing or quitting alcohol or drug use. Many women identified making new friends outside of these communities as an important support in their wellness. Specific resources mapped by women included “methadone” programs, “needle exchange”, “shelters” and temporary “housing”, substance use “treatment”, “social workers”, “prenatal classes”, community support programs for at-risk mothers and their children and just general “community support”.

### Strength and hope

While acknowledging the impacts of the various factors outside of their control in their lives, throughout the mapping process women also described ways that they had taken charge of their own and their family’s wellness and life outcomes. One of the most powerful examples of this commitment to self-determination was when women placed the button “stop the cycle” in the centre of their maps and talked hopefully about stopping experiences of harm and addiction with their own children. The button “inner strength” was also placed near the center of most women’s maps:

And strength is one of the biggest ones because out of all … out of the fear … hope … like, I could say a lot of them … violence, stress, partners even. There’s always strength to your weaknesses. And if you remember those weaknesses, you could find a better strength to [get] through it again. (Francesca)

Strength was often placed next to the “pride and dignity” button on women’s maps, which was described as something they were striving towards, either through finding a job, going back to school or “doing something that my kids can look up to”. For others, it was something they had already done, through establishing a strong sense of identity, providing for their families and maintaining their sobriety, their employment or their schooling.

Most women placed the “hope” button near the center of their maps, as either something that has supported them, or as something they needed in their lives. As Winifred, a mother of two, in her mid-thirties explained as she placed “hope” on her inner circle, what she needed to reduce her substance use was “to have something to look forward to”, such as the possibility of regaining full custody of her daughter (which she currently shared with her own father). For many women, their future hopes and dreams revolved around their children, either through the hope of regaining custody of them or for having the opportunity to help them lead happy and safe lives. Some of them discussed how staying positive and looking to the future were often difficult due to the multiple obstacles and challenges they saw between where they were and where they wanted to be. Many women talked about the need to persevere and to fight to achieve wellness for themselves and their families: “I never stopped

believing and I just … and somehow, someway … things are slowly falling into place now in my life … you know. It might not be coming … you know … real fast but there’s [hope]” (Cecile, mother of three, in her late-twenties currently in substance use treatment program).

Women expressed feelings of accomplishment and growth, through completion of treatment programs or securing safe housing, spending time with their children and providing financially for their families.

### Mothering

Throughout the mapping process, women’s children and their roles as mothers appeared to be central to understanding patterns of substance use and health from women’s perspectives. Regardless of having custody or not of their children, almost all women put their children at the center of their maps, because they saw their children as “all my life” and talked about how their love and bond with their children supported their overall wellness. The biggest detractor from their overall health was losing custody of their children or not being able to see them, either through lost visitation rights or as a self-imposed rule to protect their children from their substance use. Often, losing their children was linked to increased substance use, as well as other detrimental events in their lives, including losing their housing and the end of relationships with their partners. Winifred reflected on how being able to spend time with her daughter through shared custody with her own father always makes her feel better:

She is really smart and I am really proud. I consider myself lucky because she’s that smart … even just being around my daughter, just like makes all the yucky feeling go away, you know … cause like we are doing a good job. I think we are. Cause I am there with her.

For all women, their children were a huge source of pride in their lives, or as Margo said “my kids are my biggest accomplishment.” All women were intent on having a better family life for their children and did what they thought was best for their family’s wellbeing. This included having an abortion, having their children live with other family or having them adopted to keep them safe and give them a good home and parents or keeping their children in their own care. For many women, starting their own family was a source of healing and closure: “I’ve never had a real family. So that’s all I wanted in my life. It’s all I ever wanted was to be part of a family. To have my own” (Sophie).

Like women’s own relationships with their mothers, for those who had their children apprehended, their bond remained unbreakable. Women positioned attacks on this bond as devastating to their wellbeing as well as that of their families. Feelings of grief, loss and hopelessness were associated with apprehensions of women’s children, which often re-opened old wounds from their own childhood experiences. Many women described losing their children as a trauma tantamount to all of the previous traumatic experiences in their lives.

Many women had a tumultuous, complicated, continuous and often forced relationship with child and family social services (CFSS). Most women placed child CFSS at least in their middle circle and in some cases in all three circles to depict how entrenched representatives of these services were in their lives. Most of them had mixed feelings about this relationship, and placing CFSS on the map did not require them to commit to them as positive or negative influence, but often represented both:

I always didn’t like the Ministry of Children’s because I always [got] apprehended and … they took care of me, but they just took me away from my family. Um hum, [they do judge me]. [But] ya they do help you. When I got cleaned up and got my own place and stuff and they gave my daughter back to me, they gave me the resources and who to turn to for help and stuff when I need it. Ya, so they do good and bad. (Winifred)

Unlike most research findings and policies in this area, which are individualistic, women saw themselves and their wellbeing as being interconnected with the wellbeing of those people they loved and who loved them, much as they saw themselves and their families at the center of intersecting determinants of health.

### The intersections of determinants

Once they had completed their CIRCLES maps, women were prompted to reflect on what they would want other people to understand about their lives. Women explained they wanted just that: understanding. They did not want people to judge them without first understanding the challenges they had faced and overcome in their lives, especially in relation to trauma. For some, making the CIRCLES map was the first time they had really thought about their lives in a holistic way.

When looking at their completed maps, many women stressed that all of the determinants on their maps influenced their overall health and wellness and their experiences with substance use and pregnancy. Specifically, they saw determinants as interconnected in their influence on their use of substances and experiences with pregnancy and mothering. When discussing different determinants in their lives, women made comments like “it all goes together” or “they’re all related to each other” to further explain the interconnectedness between different influences in their lives.

## Discussion

The findings of this study provide one of the first detailed descriptions of Indigenous women’s perspectives on social determinants of their health. Asking young pregnant-involved Indigenous women to map social determinants of substance use based on their own experiences provided a more nuanced and complete understanding of social contextual factors contributing to substance use than current models of social determinants of health. In addition, women identified determinants that both contributed to and detracted from their overall wellness. They were also able to demonstrate the interconnected and complex relationships among the determinants of substance use.

The notion of nested, relational and intersectional relationships is a predominant feature of the ILCSD model (Reading & Wien, 2009) and the design of the CIRCLES map was informed by this notion. Many women commented on how helpful, eye-opening or “cool” it was to see all of the influences in their lives as they reflected on the inter-related and non-linear determinants in the maps they developed. Most women created their maps with the outer circle depicting things that were part of their history or their past, including negative influences in their lives, the middle circle depicting supports or obstacles in their lives and the inner circle representing current individual-level factors influencing their lives or their hopes for the future. When considering the emphasis on intersectionality in understanding differentially lived social inequalities among people (de Leeuw & Greenwood, 2011), this temporal element of women’s understandings of the intersections between different determinants in their lives seems appropriate. Women’s stories highlighted the relevance of intersectionality-based analysis to reveal the multiple social locations and power influences that impacted their life experiences, while also pointing to the importance of approaching research from a post-colonial perspective. The contemporary social conditions of inequality for the participants were entrenched in ongoing traumatic events and, as shown in previous research findings, were also related to assimilatory policies that led to the loss of land, the decades of incarceration of Aboriginal children and the high levels of child sexual abuse, sexual assault and domestic violence in many Aboriginal communities (Haskell & Randall, 2009).

Models describing the social determinants of health for Indigenous peoples have been proposed. One of the most comprehensive models is the ILCSD model (Reading & Wien, 2009). The findings of this study point to limitations in the ILCSD. It does not accommodate women’s roles as women, partners and mothers and the subsequent influences on their experiences of substance use and pregnancy. As shown in these findings, pervasive experiences of sexual abuse and violence throughout their lives influenced women’s efforts to fulfill responsibilities as protectors and leaders in their family, their deferral of resources and opportunities for the benefit of their families, partners and/or children and their roles as the often sole-parent. Failure to acknowledge these women-centered determinants is a major limitation of previous models and understandings of the contexts of substance use. A women-centered approach to health (Fahy, 2012), on the other hand, is informed by women’s lives and experiences, places them at the center of services and activities and works to eliminate gendered health inequalities. Such an approach is essential to understanding the social determinants of substance use among young Indigenous women in Canada.

While women created their CIRCLES maps, they identified not only factors that contributed to their substance use and negative life experiences, but also focused on the factors that helped them heal, supported their reduction or cessation of alcohol or drug use and contributed to their own and their family’s overall wellness. Women highlighted their goals for their futures and most saw their final CIRCLES map as a type of road-map from where they had been to where they wanted to be. Overall, regardless of what each woman’s current situation was, in creating their CIRCLES maps their approach was hopeful, in contrast to prevailing pathologizing portrayals of Indigenous peoples and their health (Allen & Smylie, 2015) and in keeping with previous research with Aboriginal women that highlighted their own experiences and perspectives (Kurtz, Nyberg, Van Den Tillaart, & Mills, 2008). These findings stand in contrast to the lack of attention to strengthening the positions of women as mothers, with most policy in this area instead favoring a deficits approach, strictly from the point of view of the rights of the child (Greaves & Poole, 2004).

The CIRCLES maps made clear that supports needed for women’s wellness are not in competition with the supports needed for the wellness of their children and their families, but rather mutually supportive. In traditional Aboriginal communities, women were the heart of the communities and this continues to be true, with many women explicitly recognizing the importance of their role in contributing to and influencing the next generation (Halseth, 2013). Others have called for efforts to support the health and wellness of young Aboriginal mothers who use substances because they are not only critical for their health as individuals, but for the revitalization of families and communities (Halseth, 2013). Importantly, as shown in this study’s findings, the scale and scope of the challenges faced by young pregnant-involved Indigenous women in Canada who use alcohol and drugs and their persistence and survival despite great odds is clear evidence of their individual and collective resilience. Accordingly, then, as opposed to focusing exclusively on vulnerability and pathology, also including a focus on resilience necessarily shifts the attention to the resources, strengths and positive outcomes in women’s lives (Kirmayer, Dandeneau, Marshall, Phillips, & Williamson, 2012) while highlighting opportunities to further foster and support resilience through policies and interventions.

By expanding research to examine and value women’s lives and experiences beyond the reproductive period, there is an opportunity to support and inform programs that address women’s needs from a holistic perspective (Nathoo et al., 2013), while countering the framing of problematic substance use through gendered responsibilization of women (Benoit et al., 2014) that leads to harmful, simplistic and morality-based conceptualizations of substance use during pregnancy. As evidenced through women’s CIRCLES maps, women’s experiences with both substance use and pregnancy occur within, across and relative to multiple and complex life contexts that require further attention. Due diligence must be paid to understanding women’s life histories beyond the reproductive period to support their health and wellness.

The findings of this study should be considered in light of its limitations. Convenience sampling was used. Many of the participants were street-entrenched and street-recruited women facing extraordinary risks in their day-to-day lives and are not representative of the larger Indigenous women population in Canada or elsewhere. Since not all women were currently pregnant at the time of interview, the data may be subject to recall bias.

The CIRCLES mapping activity was a novel approach used in this study to generate data. Although the CIRCLES map provided structure that may have influenced or limited women’s abilities to fully represent their experiences, the variability in the patterns evident in the way women completed the task suggests that the structure allowed for some flexibility.

Increasingly, there has been a call for interventions and practices designed to foster and enhance the health and wellbeing of Aboriginal families to engage with holistic concepts of health, moving beyond the bio-medical realms to instead address and focus on the social determinants (Greenwood & De Leeuw, 2012). This research project makes clear the importance of addressing substance use among pregnant-involved young Aboriginal women from a social determinants of health perspective. More specifically, it provides support for including Aboriginal-specific determinants of health when looking at health issues among young Aboriginal women. In conclusion, the perspectives of young pregnant-involved Indigenous women in Canada are paramount to understanding the social determinants of substance use from a woman-informed perspective. By honoring women’s voices and life experiences, effective, appropriate and respectful programs that support young pregnant-involved Indigenous women in Canada’s wellness, strength and resilience can be developed.

## Notes

1. Collectively refers to the original people of North America, including the three distinct groups of First Nations (historically referred to as Indian), Metis and Inuit peoples (Constitution Act, 1867). Over 1.4 million individuals in Canada identify themselves as Aboriginal (Statistics Canada, 2012).

2. The terms Aboriginal and Indigenous are used in accordance with the term used by participants in the study or the cited authors. Where not specified, the authors have chosen to use the term Indigenous.

3. Refers to Aboriginal peoples globally (Indigenous and Northern Affairs Canada, 2012).
